# Efficacy of a Large Language Model Data Extraction System in Evidence Reviews for Emerging Infectious Diseases: A Randomized Crossover Trial

**DOI:** 10.1093/ofid/ofag401

**Published:** 2026-07-23

**Authors:** Masahiro Ishikane, Yuki Kataoka, Yasushi Tsujimoto, Yuki Moriyama, Yukimasa Matsuzawa, Norio Ohmagari

**Affiliations:** Disease Control and Prevention Center, National Centre for Global Health and Medicine, Japan Institute for Health Security, Shinjuku, Tokyo, Japan; Center for Postgraduate Clinical Training and Career Development, Nagoya University Hospital, Nagoya, Aichi, Japan; Center for Medical Education, Graduate School of Medicine, Nagoya University, Nagoya, Aichi, Japan; Scientific Research Works Peer Support Group (SRWS-PSG), Osaka, Japan; Department of Internal Medicine, Kyoto Min-Iren Asukai Hospital, Kyoto, Japan; Department of Healthcare Epidemiology, Kyoto University Graduate School of Medicine/School of Public Health, Kyoto, Japan; Department of International and Community Oral Health, Tohoku University Graduate School of Dentistry, Sendai, Miyagi, Japan; Scientific Research Works Peer Support Group (SRWS-PSG), Osaka, Japan; Oku Medical Clinic, Osaka, Japan; Department of Health Promotion and Human Behavior, Kyoto University Graduate School of Medicine/School of Public Health, Kyoto University, Kyoto, Japan; Division of Rheumatology, Department of Internal Medicine, Showa University School of Medicine, Shinagawa, Tokyo, Japan; Disease Control and Prevention Center, National Centre for Global Health and Medicine, Japan Institute for Health Security, Shinjuku, Tokyo, Japan; Disease Control and Prevention Center, National Centre for Global Health and Medicine, Japan Institute for Health Security, Shinjuku, Tokyo, Japan; Disease Control and Prevention Center, National Centre for Global Health and Medicine, Japan Institute for Health Security, Shinjuku, Tokyo, Japan

**Keywords:** artificial intelligence, emerging infections, large language model, randomized crossover trial

## Abstract

**Background:**

Rapid evidence synthesis during emerging infectious and re-emerging disease outbreaks is critical, yet traditional systematic reviews rarely meet urgent timelines. Large language models (LLMs) may accelerate evidence synthesis by extracting data from publications. We compared an LLM-assisted data extraction system with manual extraction.

**Methods:**

We conducted a 1:1, open-label, 2-period, randomized crossover trial at the National Center for Global Health and Medicine, a national reference center for emerging infectious diseases in Japan (2025). Five experienced reviewers extracted predefined items from mpox-related articles under 2 conditions: (i) LLM-assisted extraction using OpenAI's o3 model to generate structured summaries and (ii) manual review of PDF files. The primary outcome was task completion time; secondary outcomes were extraction accuracy and adverse events. Mixed-effects models included condition as a fixed effect and participant and paper IDs as random effects. The protocol, source code, and data are available at https://github.com/SRWS-PSG/emerging_infection_24K13518_open

**Results:**

Five evaluators (4 physicians and 1 pharmacist; 6–10 years postgraduation) completed 20 task-level evaluations (LLM, n = 9; no LLM, n = 11). Mean completion time was 27.5 minutes with LLM assistance versus 34.5 minutes without. The LLM-assisted condition was 7.9 minutes faster on average (95% CI −1.5 to 17.3; *P* = .099). Extraction accuracy was 100% in both conditions, and no adverse events were reported.

**Conclusions:**

LLM assistance might reduce data extraction time by ∼23% (7.9 minutes per article; 95% CI −1.5 to 17.3 minutes) with no observed loss of accuracy. Although statistical uncertainty remains, LLM integration may offer practical value for rapid evidence synthesis during public health emergencies as tools and prompting strategies mature.

## Teaser

LLM-assisted data extraction in a randomized crossover trial reduced completion time by about 23% (7.9 minutes per article; 95% CI −1.5 to 17.3 minutes) versus manual review while maintaining 100% accuracy and no adverse events. Although statistical uncertainty remains, the results suggest practical benefits for rapid evidence synthesis during outbreaks.

Rapid and accurate information, evidence collection, and analysis are critical for public health decision-making during emerging infectious disease outbreaks. In emergency situations such as the COVID-19 and mpox pandemics, there is an urgent need to provide evidence expeditiously; however, traditional systematic review methodologies often fail to meet these time constraints [1]. Rapid evidence synthesis is essential to enable policymakers to make the necessary decisions such as notification, contact tracing, strengthening testing and medical systems, and securing medical countermeasures within short timeframes, and the development of tools that can swiftly collect and synthesize data has become imperative [2].

The application of automated technologies, particularly large language models (LLMs), has demonstrated the potential to address efficiency challenges in emergent evidence synthesis. Automated data extraction that leverages LLM is expected to enhance both the speed and accuracy of the review process [3, 4]. Implementing these technologies may enable the rapid synthesis and delivery of evidence while minimizing human resource requirements, potentially facilitating timely information provision to decision makers.

However, empirical data demonstrating the extent to which these tools reduce the actual burden of evidence synthesis are lacking. We developed an LLM-based application that extracted structured data from articles in PDF format [5–8]. The objective of this randomized crossover trial was to evaluate whether the use of an LLM-based system improves the time efficiency of extracting data from articles on emerging infectious diseases compared with manual extraction without such a system.

## METHODS

### Patient Consent Statement

The study was registered in the UMIN Clinical Trials Registry (UMIN000058346). The research protocol was exempted from review by the Institutional Review Board for Clinical Research of the Japan Institute for Health Security because the study evaluated professional efficiency and involved no medical interventions or patient health outcomes.

### Trial Design

**Figure 1.**
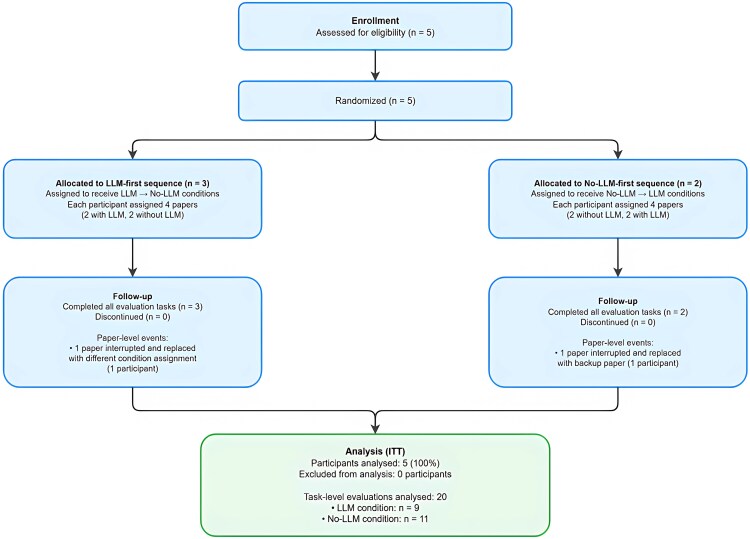
Enrollment Process. This study was a 1:1 open-label, 2-period, 2-group, randomized crossover trial with a superiority framework designed to evaluate the efficacy of a LLM-based data extraction system.

This study was a 1:1 open-label, 2-period, 2-group, randomized crossover trial with a superiority framework designed to evaluate the efficacy of a LLM-based data extraction system. This study was conducted in accordance with the CONSORT guidelines [9]. Details are presented in Figure 1 and Supplementary Table 1.

### Study Populations

This study was conducted at the National Center for Global Health and Medicine (NCGM), a national reference center for emerging infectious diseases in Japan. We recruited researchers and healthcare professionals whose native language is Japanese from the center through internal emails, research meeting announcements, and personal invitations. Eligible participants had experience in evidence reviews and specialization in infectious diseases, possessed basic computer skills, and provided informed consent. We excluded individuals with unstable internet connections. The sample size was calculated to detect an expected 10-minute difference in task completion time with 80% power and a 5% significance level, requiring 20 participant-paper evaluations. We recruited 5 participants, each assigned to evaluate 4 papers in August 2025.

### Intervention and Control

The intervention group used an LLM-based system to automatically extract and summarize structured data from research paper PDFs using OpenAI's o3 model (OpenAI, San Francisco, CA; accessed 14 October 2025) via OpenAI Application Programming Interface (API). The participants reviewed the system-generated summary to complete the data entry web form. The control group did not use the LLM system; instead, they read the paper PDFs directly and manually extracted the information to complete the same web form.

### Randomization

The study statistician randomized the participants into 2 sequences using a computer-generated random number table. We used block randomization with a block size of 2 to ensure a 1:1 allocation ratio. Allocation was concealed because the statistician had no contact with the participants, and the web application assigned the intervention immediately before the task, preventing participants or enrolling staff from predicting the sequence.

### Procedure

After obtaining electronic informed consent and confirming eligibility, each participant was assigned a paper. The web application then assigned the participants to either the intervention or control condition for that task. The intervention group received the LLM-generated summary, whereas the control group received only the paper PDF about mpox-related articles [5–8]. For the intervention materials, PDF text was extracted with pdfminer.six and submitted to the OpenAI o3 model via the API. The first 10 000 characters of each PDF text were used as input. The system prompt instructed the model to act as an expert in extracting structured data from research papers, and the user prompt asked the model to extract structured information from the provided paper text. Responses were constrained using a strict JSON schema requiring filename, theme, category, time, place, person, and a 3–5 item Japanese plain-language bullet-point summary. No task-specific training, fine-tuning, or few-shot examples were used. The temperature parameter was not set because it was not supported by the o3 model in our implementation. Each paper was processed once before the trial, and the resulting summaries were stored as fixed intervention materials. We have completed TRIPOD-LLM checklist as reporting checklists (Supplementary Table 2).

Limiting the scope to peer-reviewed English-language articles, the first author selected key literature that the 5 reviewers were not expected to have read in detail previously. Both groups completed a data entry form with fields for paper summaries, assessments, and proposed clinical actions. The data extraction process consists of 5 steps: downloading the article, summarizing the paper (with optional LLM assistance), assessing the article, proposing a clinical action, and submitting completed information. The LLM is used only for summarizing articles and is not used for making assessments or proposed action. In the non-LLM group, the use of translation software was permitted, but the use of artificial intelligence (AI) tools was not. The system automatically recorded the time from the start of data entry to submission. Each participant repeated this process for all 4 assigned papers, according to their randomized sequences.

### Outcomes

The prespecified primary outcome was the task completion time, measured in minutes from the start of data entry to form submission. Task completion time was selected as the primary outcome because the fundamental challenge driving this study is the urgent need for rapid evidence synthesis during emerging infectious disease outbreaks, where time efficiency is the primary determinant of practical utility. This approach is consistent with recent LLM-assisted evidence synthesis studies that have similarly prioritized time efficiency as a key evaluative dimension [3, 10]. Prespecified secondary outcomes were data extraction accuracy and harm. Data extraction accuracy was defined as the proportion of correct answers as assessed by an expert reviewer. A single infectious disease specialist (the first author) evaluated the content of the article summaries. The evaluation focused on verifying the accuracy of the intended meaning of the articles, rather than the verbatim translation. The evaluator was unaware of group allocation. Also, we developed the AI-assisted English translations for detailed inspection of the original responses in the Supplementary Table 3. Harms were defined as nonmedical adverse events, such as excessive psychological stress or significant task difficulty, and they were monitored through voluntary participant reporting.

### Statistical Analysis

We analyzed the primary outcome using a linear mixed-effects model, with the intervention as a fixed effect and the participant and paper IDs as random effects. We analyzed the accuracy of data extraction using a generalized linear mixed model. Secondary outcomes were summarized descriptively. All analyses followed the intention-to-treat principle and included all randomized participants. Statistical analyses were performed using Python (version 3.11.0). The source code is available at https://github.com/SRWS-PSG/emerging_infection_24K13518_open

## RESULTS

### Background Information on Evaluators and Articles

The mean number of postgraduate years among the 5 evaluators (4 medical doctors and 1 pharmacist) was 7.8 years (range: 6–10 years). Six papers (3 epidemiology, 2 laboratory, and 1 vaccine study) contained a mean of 3365 words per paper excluding references (range: 2155–5588 words).

### Primary Outcomes

**Figure 2.**
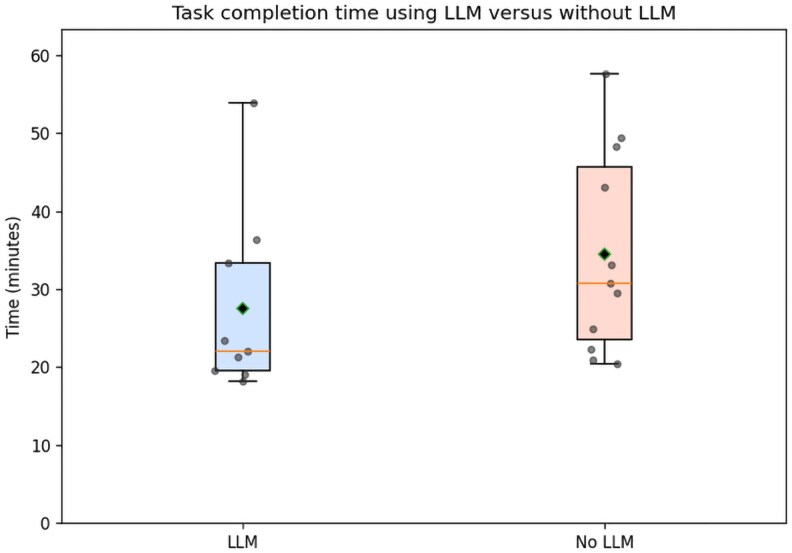
Task completion time using LLM versus without LLM. Figure 2 shows the task completion time by condition. Boxplots summarize task-level completion times for the LLM and no LLM conditions (n = 9 and n = 11 task evaluations, respectively). Boxes show the interquartile range (IQR), horizontal lines denote medians, and whiskers indicate the range of observed values. Green diamonds mark means. Gray points represent individual task observations with small horizontal jitter for visibility. The *y*-axis is in minutes.

Across 20 task-level evaluations (LLM: n = 9; no LLM: n = 11), the mean time to complete the data extraction task was 27.5 minutes with LLM assistance and 34.5 minutes without LLM assistance. In a linear mixed-effects model with participant and paper as random effects, the LLM condition was, on an average, 7.9 minutes faster than the no LLM condition (95% CI for the reduction, −1.5 to 17.3; *P* = .099). Although the point estimate favored LLM use, the confidence interval included zero (Figure 2).

### Secondary Outcomes

Supplementary Table 3 provides the raw extraction data and their AI-assisted English translations for detailed inspection of the original responses. Based on expert review of the intended meaning of each extracted item, accuracy was 100% in both conditions (LLM 9/9; no LLM 11/11). Comparative modeling was not estimable because there was no between-group variability. No harm or adverse events, as prespecified (eg, excessive psychological stress or significant task difficulty), were reported by participants in either condition during the study.

## DISCUSSION

This randomized crossover trial evaluated the time efficiency and accuracy of LLM-assisted data extraction from literature on emerging infectious diseases compared with manual extraction. Our findings demonstrated that LLM assistance reduced the mean task completion time by 7.9 minutes (27.5 minutes vs 34.5 minutes), representing an approximately 23% reduction in extraction time. However, this difference did not reach statistical significance (95% CI: −1.5 to 17.3 minutes; *P* = .099). Notably, the data extraction accuracy was perfect in both conditions (100%), and no adverse events or participant burden was reported throughout the study, suggesting that LLM implementation is safe and maintains data quality standards.

Automation, including LLM, is being increasingly explored to reduce the time and effort involved in evidence synthesis; however, its adoption and reporting practices remain limited. Based on a study conducted between 2017 and 2024 [11], only ∼5% of the studies explicitly reported using ML, with most applications limited to screening tasks among the 2271 articles. In this study, we evaluated the effectiveness of summarization using an LLM-based system. AI-assisted tools show promise in improving workflow efficiency in clinical documentation. One study evaluated the impact of an AI scribe on clinician documentation efficiency and observed modest but significant improvements [12]. In pre–post comparisons among 125 users, median electronic health records time per encounter decreased by 2.0 minutes, note-writing time by 0.5 minutes, and time to close notes by 7.1 hours. In the adjusted between-group analyses, AI scribe users had 8.5% less electronic health record time (2.4 minutes per encounter) and 15.9% less note-writing time (1.8 minutes) than nonusers. These results indicate that AI scribes can meaningfully reduce the documentation burden, even though the effects on after-hours work and visit volumes are minimal. In our study, although LLM support did not demonstrate a statistically significant difference, the average task completion time was reduced by 7.9 minutes (27.5 minutes vs 34.5 minutes).

Although the application of LLMs to automate systematic review processes has gained considerable attention, research on LLM-assisted data extraction, specifically in living systematic reviews, remains limited [13]. Collaborative LLM approaches can achieve high accuracy (94%) in extracting data from clinical trial publications [13]; however, these investigations have predominantly focused on oncology trials rather than infectious diseases. Notably, to the best of our knowledge, no prior randomized controlled trial has evaluated the time efficiency of LLM-assisted data extraction in the context of emerging infectious diseases where rapid evidence synthesis is critical [10]. Our study addresses this gap by directly measuring the time savings achieved through LLM implementation and assessing the data accuracy in real-time extraction scenarios relevant to public health emergencies.

The findings of this study have important implications for future evidence synthesis of emerging infectious diseases. The 7.9 minutes reduction in task completion time suggests meaningful practical benefits. Importantly, the developed LLM-assisted system is fully accessible, the source code is openly available, and implementation requires only a Google account and API access, making it potentially deployable by research teams worldwide. However, practical adoption should be considered in light of technical barriers because successful implementation requires proficiency in programming, API integration, and prompt engineering. Despite these skill requirements, the 100% accuracy maintained across both conditions and the absence of adverse events suggest that, when properly implemented, LLM-assisted extraction can serve as a safe and reliable tool to accelerate evidence synthesis without compromising data quality. This is particularly valuable in pandemic settings, where even modest time savings can expedite critical public health decision-making.

Several directions for future research have emerged from this study. First, the applicability of this approach to fields other than emerging infectious diseases requires further investigation. The workflow we developed may be adapted for rapid evidence synthesis in other rapidly evolving medical domains, such as oncology clinical trials or novel therapeutic interventions. Second, our study was conducted with participants who were familiar with the literature review methodologies and possessed baseline expertise in data extraction. Since the evaluators for this study were recruited from the NCGM, national center for emerging infectious diseases in Japan, it is possible that relatively experienced evaluators were selected in this study. Such experienced evaluators are likely already skilled at quickly identifying and extracting key information from articles, which may have diminished the relative advantage offered by the prestructured summaries generated by LLM. On the other hand, if less experienced evaluators had participated in this study, they might have benefited more from the support of the LLM, as reported in previous literature [10, 14], which could have led to an underestimation of the LLM's potential time-saving benefit. Future studies should evaluate the performance of this LLM system among users who lack domain-specific knowledge of infectious diseases and experience with systematic literature extraction. Such research would clarify whether LLM assistance can democratize evidence synthesis by enabling less experienced researchers or nonspecialists to conduct high-quality data extraction or whether technical and domain expertise remain prerequisites for effective implementation. By enabling system construction with fewer experts, experts can focus only on tasks they can perform, such as clinical management. Moreover, relevant examples outside medicine include LLM-supported data extraction in software engineering systematic mapping studies [15], hypothesis-evidence classification in the social sciences [16], and structured extraction from educational and materials-science literature [17]. These studies suggest that LLMs may support semi-automated evidence synthesis workflows, although human verification remains essential.

This study had several limitations. First, our sample size was relatively small (n = 20 task-level evaluations), which may have contributed to the lack of statistical significance, despite the observed time reduction. The study population was limited to individuals with expertise in literature review methodologies and data extraction, potentially limiting the generalizability of our findings to less experienced users or those without domain-specific knowledge of infectious diseases. Second, our LLM-assisted system requires the manual downloading of PDF files, indicating that full automation of the evidence synthesis workflow has not yet been achieved. This intermediate step requires human intervention and may limit the scalability of this approach in high-throughput scenarios. Third, the current study focused exclusively on data extraction and did not evaluate the performance of the LLM system in upstream processes such as literature search, screening, or article selection. These critical components of systematic reviews remain outside the scope of our validation, and their automation requires further investigation. Fourth, the study was powered to detect a 10-minute difference; however, the observed difference was 7.9 minutes with greater within-group variability than assumed, suggesting the study may have been underpowered to detect an effect of this magnitude. The result should therefore be interpreted with caution. Finally, we did not formally assess output stability under repeated prompting. However, potential run-to-run variability did not affect the trial exposure because all LLM summaries were generated before participant sessions and stored as fixed materials. Future studies should evaluate repeated-prompt stability and report model-version-specific reproducibility.

In conclusion, this randomized controlled trial demonstrated that infectious disease specialists sing an LLM-assisted data extraction system achieved a modest reduction in task completion time while maintaining perfect data accuracy. Although statistical uncertainty remains, the combination of time efficiency and preserved data quality suggests that LLM-assisted systems may offer practical value to infectious disease specialists for conducting rapid evidence synthesis during public health emergencies. As technology continues to evolve and researchers gain experience with prompt engineering and system optimization, LLM-assisted data extraction may become an increasingly valuable component of the infectious disease evidence synthesis toolkit, particularly in time-sensitive scenarios where even modest efficiency gains can meaningfully impact public health responses.

## Supplementary Material

ofag401_Supplementary_Data

## Supplementary Data

Supplementary materials are available at *Open Forum Infectious Diseases* online. Consisting of data provided by the authors to benefit the reader, the posted materials are not copyedited and are the sole responsibility of the authors, so questions or comments should be addressed to the corresponding author.
