# Supplementary material for: Efficacy of a Large Language Model Data Extraction System in Evidence Reviews for Emerging Infectious Diseases: A Randomized Crossover Trial
Source: Open Forum Infect Dis. 2026 Jul 23;13(7):ofag401. doi: 10.1093/ofid/ofag401 (PMC13393321; doi:10.1093/ofid/ofag401)
Supplement: ofag401_Supplementary_Data [file ofag401_supplementary_data.zip › Supplemental_Table 1.docx]

# Supplemental Table 1. CONSORT 2025 Checklist - Filled Version

**Manuscript:** Efficacy of a large language model data extraction system in evidence reviews for emerging infectious diseases: A randomized crossover trial

**Date:** 11 January 2026

|  | Section/topic | No | CONSORT 2025 checklist item description | | Reported in section |
| --- | --- | --- | --- | --- | --- |
|  | **Title and abstract** |  |  | |  |
|  | Title and structured abstract | 1a | Identification as a randomised trial | | Title |
|  |  | 1b | Structured summary of the trial design, methods, results, and conclusions | | Abstract (Background, Materials, Results, Conclusions) |
|  | **Open science** |  |  | |  |
|  | Trial registration | 2 | Name of trial registry, identifying number (with URL) and date of registration | | Methods - Ethics Approval (UMIN000058346) |
|  | Protocol and statistical analysis plan | 3 | Where the trial protocol and statistical analysis plan can be accessed | | Materials (GitHub URL: https://github.com/SRWS-PSG/emerging_infection_24K13518_open) |
|  | Data sharing | 4 | Where and how the individual de-identified participant data (including data dictionary), statistical code and any other materials can be accessed | Methods - Statistical Analysis (GitHub URL + data available upon reasonable request) | |
|  | Funding and conflicts of interest | 5a | Sources of funding and other support (eg, supply of drugs), and role of funders in the design, conduct, analysis and reporting of the trial | Funding source (JSPS KAKENHI JP24K13518, 25K13447, 25K13585; funders played no role) | |
|  |  | 5b | Financial and other conflicts of interest of the manuscript authors | Conflict of interest (no conflicts declared) | |
|  | **Introduction** |  |  |  | |
|  | Background and rationale | 6 | Scientific background and rationale | Introduction (paragraphs 1–2) | |
|  | Objectives | 7 | Specific objectives related to benefits and harms | Introduction (final paragraph) | |
|  | **Methods** |  |  |  | |
|  | Patient and public involvement | 8 | Details of patient or public involvement in the design, conduct, and reporting of the trial | Patient and public involvement | |
|  | Trial design | 9 | Description of trial design including type of trial (eg, parallel group, crossover), allocation ratio, and framework (eg, superiority, equivalence, non-inferiority, exploratory) | Methods - Trial design | |
|  | Changes to trial protocol | 10 | Important changes to the trial after it commenced including any outcomes or analyses that were not prespecified, with reason | Changes to trial protocol | |
|  | Trial setting | 11 | Settings (eg, community, hospital) and locations (eg, countries, sites) where the trial was conducted | Methods - Trial design, and Study Populations | |
|  | Eligibility criteria | 12a | Eligibility criteria for participants | Methods - Study Populations | |
|  |  | 12b | If applicable, eligibility criteria for sites and for individuals delivering the interventions (eg, surgeons, physiotherapists) | Methods - Procedure (not applicable) | |
|  | Intervention and comparator | 13 | Intervention and comparator with sufficient details to allow replication. |  | |
|  | Outcomes | 14 | Prespecified primary and secondary outcomes, including the specific measurement variable (eg, systolic blood pressure), analysis metric (eg, change from baseline, final value, time to event), method of aggregation (eg, median, proportion), and time point for each outcome | Methods - Outcomes | |
|  | Harms | 15 | How harms were defined and assessed (eg, systematically, non-systematically) | Methods - Outcomes | |
|  | Sample size | 16a | How sample size was determined, including all assumptions supporting the sample size calculation | Methods - Trial design | |
|  |  | 16b | Explanation of any interim analyses and stopping guidelines | Methods - Statistical Analysis | |
|  | Randomisation: |  |  |  | |
|  | Sequence generation | 17a | Who generated the random allocation sequence and the method used | Methods - Randomization | |
|  |  | 17b | Type of randomisation and details of any restriction (eg, stratification, blocking and block size) | Methods - Randomization | |
|  | Allocation concealment mechanism | 18 | Mechanism used to implement the random allocation sequence (eg, central computer/telephone; sequentially numbered, opaque, sealed containers), describing any steps to conceal the sequence until interventions were assigned | Methods - Randomization | |
|  | Implementation | 19 | Whether the personnel who enrolled and those who assigned participants to the interventions had access to the random allocation sequence | Methods - Procedure | |
|  | Blinding | 20a | Who was blinded after assignment to interventions (eg, participants, care providers, outcome assessors, data analysts) | Methods - Trial design | |
|  |  | 20b | If blinded, how blinding was achieved and description of the similarity of interventions | N/A | |
|  | Statistical methods | 21a | Statistical methods used to compare groups for primary and secondary outcomes, including harms | Methods - Statistical Analysis | |
|  |  | 21b | Definition of who is included in each analysis (eg, all randomised participants), and in which group | Methods - Statistical Analysis | |
|  |  | 21c | How missing data were handled in the analysis | Results - Background information on evaluators and articles | |
|  |  | 21d | Methods for any additional analyses (eg, subgroup and sensitivity analyses), distinguishing prespecified from post hoc | Methods - Statistical Analysis | |
|  | **Results** |  |  |  | |
|  | Participant flow, including flow diagram | 22a | For each group, the numbers of participants who were randomly assigned, received intended intervention, and were analysed for the primary outcome | Results - Background information on evaluators and articles | |
|  |  | 22b | For each group, losses and exclusions after randomisation, together with reasons | Results - Background information on evaluators and articles | |
|  | Recruitment | 23a | Dates defining the periods of recruitment and follow-up for outcomes of benefits and harms | Methods-Trial design | |
|  |  | 23b | If relevant, why the trial ended or was stopped | N/A | |
|  | Intervention and comparator delivery | 24a | Intervention and comparator as they were actually administered (eg, where appropriate, who delivered the intervention/comparator, how participants adhered, whether they were delivered as intended [fidelity]) | Results - Background information on evaluators and articles | |
|  |  | 24b | Concomitant care received during the trial for each group | N/A | |
|  | Baseline data | 25 | A table showing baseline demographic and clinical characteristics for each group | Results - Background information on evaluators and articles | |
|  | Numbers analysed, outcomes and estimation | 26 | For each primary and secondary outcome, by group: ● the number of participants included in the analysis ● the number of participants with available data at the outcome time point ● result for each group, and the estimated effect size and its precision (such as 95% confidence interval) ● for binary outcomes, presentation of both absolute and relative effect size | Results - Secondary Outcomes | |
|  | Harms | 27 | All harms or unintended events in each group | Results - Secondary Outcomes | |
|  | Ancillary analyses | 28 | Any other analyses performed, including subgroup and sensitivity analyses, distinguishing pre-specified from post hoc | N/A | |
|  | **Discussion** |  |  |  | |
|  | Interpretation | 29 | Interpretation consistent with results, balancing benefits and harms, and considering other relevant evidence | Discussion | |
|  | Limitations | 30 | Trial limitations, addressing sources of potential bias, imprecision, generalisability, and, if relevant, multiplicity of analyses | Discussion | |

N/A: not applicable

**Citation:** Hopewell S, Chan AW, Collins GS, et al.  CONSORT 2025 statement: Updated guidelines for reporting randomized trials. BMJ. 2025; 388:e081123. https://dx.doi.org/10.1136/bmj-2024-081123
