# Supplementary material for: Efficacy of a Large Language Model Data Extraction System in Evidence Reviews for Emerging Infectious Diseases: A Randomized Crossover Trial
Source: Open Forum Infect Dis. 2026 Jul 23;13(7):ofag401. doi: 10.1093/ofid/ofag401 (PMC13393321; doi:10.1093/ofid/ofag401)
Supplement: ofag401_Supplementary_Data [file ofag401_supplementary_data.zip › Supplemental_Table 3.docx]

# Supplemental Table 3. Comparison of summaries generated with and without LLM assistance*

| **Translated English data with LLM** | **Translated English data without LLM** |
| --- | --- |
| ID 1-Ref 5   - During the multinational mpox outbreak in 2022, the antiviral drug tecovirimat was used on a large scale for the first time in the United States. - Homology analysis of the MPXV F13L gene identified 11 known and 13 novel resistance-associated mutations. - Drug susceptibility testing was performed on 124 specimens isolated from 68 individuals, and resistance was confirmed in 96 specimens from 46 individuals. - The phenotype of resistant strains was determined using a viral cytopathic effect assay. - Resistant strains were mainly detected in severely immunocompromised patients who had received multiple courses of tecovirimat treatment, whereas viruses from treatment-naïve patients were mostly susceptible. - Compared with the total number of patients treated with tecovirimat, the proportion of resistant viruses remained relatively low, at less than 1%. | ID 3- Ref 5   - During the multinational mpox outbreak in 2022, the antiviral drug tecovirimat was used for treatment. - The F13L gene is the target of tecovirimat, and single amino acid substitutions in F13 are known mechanisms of tecovirimat resistance. Genomic analysis identified 13 additional mutations in addition to 11 previously known mutations. - In this study, testing of 124 isolates obtained from 68 patients showed a resistant phenotype in 96 isolates from 46 patients. The factor most strongly associated with resistance was a history of multiple courses of tecovirimat treatment in the setting of severe immunodeficiency. - In contrast, viruses from patients without prior tecovirimat treatment remained susceptible. Overall, the frequency of resistance was low, at less than 1%. |
| ID 2-Ref 5   - During the multinational mpox outbreak in 2022, the antiviral drug tecovirimat was used on a large scale for the first time in the United States, in at least 7,563 patients. - Analysis of mutations in the viral F13L gene identified 11 known and 13 novel resistance-associated mutations; however, the effects of seven of the novel mutations remain unknown. - Drug susceptibility testing was performed on 124 specimens isolated from 68 individuals, and resistance was confirmed in 96 specimens from 46 individuals. - Resistant strains were mainly detected in severely immunocompromised patients who had received multiple courses of treatment, whereas viruses from treatment-naïve patients were mostly susceptible. - Compared with the total number of treated patients, the proportion of resistant viruses remained low, at less than 1%. | ID 4-Ref 5   - This article summarizes how tecovirimat, an antiviral drug, was administered on a large scale for the first time in the United States during the 2022 multinational outbreak of monkeypox virus (MPXV) infection, and how its use affected MPXV drug susceptibility. - The study was conducted by analyzing genome sequences completed at the CDC and investigating genetic mutations associated with drug susceptibility. - Among 46 patients with tecovirimat-resistant isolates, longitudinal sampling was performed in four patients. Specimens obtained before tecovirimat treatment were susceptible, whereas subsequent specimens showed resistance, indicating that tecovirimat resistance can emerge during antiviral therapy. - Characteristics of patients with resistant isolates included uncontrolled HIV infection, very low CD4+ T-cell counts, and exposure to tecovirimat during hospitalization. - These data may help inform decision-making regarding future tecovirimat use. However, it should be noted that submission of specimens for tecovirimat susceptibility testing was voluntary, and specimen collection may have been biased toward particular physicians, hospitals, or geographic regions. |
|  | ID 5-Ref 5   - TPOXX, a therapeutic agent containing tecovirimat, was approved in the United States in 2018 for the treatment of smallpox. - Since May 2022, tecovirimat was administered to at least 7,563 patients, and 435 patients with suspected tecovirimat resistance underwent testing for resistance. Among specimens obtained from 68 patients that underwent both genetic and phenotypic testing, a resistant phenotype was confirmed in 46 patients. - In this study, genome-sequenced MPXV was used to infect cells, isolated, and then cultured in cells treated with tecovirimat. Using a cytopathic effect assay, the investigators showed that isolates harboring the E353K mutation retained susceptibility to tecovirimat. - A total of 130 specimens were collected from 76 patients, and mutations in the F13 protein were examined. This analysis identified 11 amino acid substitutions associated with a resistant phenotype. - A resistant phenotype was observed in 83 isolates from 41 patients, and a partially resistant phenotype was observed in 16 isolates from 11 patients; however, the clinical significance of these findings remains unclear. - In 4 of the 46 patients, resistance that was absent before tecovirimat administration emerged after treatment. - Resistance-associated mutations were detected in patients without a history of tecovirimat treatment, suggesting the possibility of transmission of resistant virus. - Among six cases in which tecovirimat resistance was suspected, one patient improved clinically; however, the remaining patients deteriorated during tecovirimat treatment and responded to cidofovir. Therefore, cidofovir should be considered when tecovirimat resistance is suspected. - Tecovirimat resistance was associated with uncontrolled HIV infection, low CD4-positive T-cell counts, and a history of tecovirimat administration during hospitalization. |
| ID 8-Ref 6   - Monkeypox virus belongs to the genus Orthopoxvirus, subfamily Chordopoxvirinae, family Poxviridae, which also includes variola virus, vaccinia virus, and cowpox virus. - These viruses are genetically and antigenically very similar, resulting in cross-protective immunity. Therefore, smallpox vaccination provides protective effects against monkeypox. - Outbreaks in African countries have occurred in areas with difficult access and in regions affected by armed conflict, and have been associated with shortages of healthcare infrastructure and healthcare workers. - Both adults and children can be infected, and the disease typically progresses through three stages: the incubation period, the prodromal phase, and the rash phase. - The mean incubation period is 13 days. The prodromal phase lasts 1–4 days and is characterized by high fever, headache, fatigue, and lymphadenopathy. - The rash phase lasts 14–28 days and eventually progresses through a pustular stage. - Patients may have anywhere from a few to several thousand lesions, which are distributed mainly on the face, trunk, arms, and legs. - Lesions frequently occur on the palms and soles, which is one of the features that distinguishes monkeypox from varicella. In addition, all lesions are usually at the same stage of development, helping to distinguish monkeypox from other diseases with cutaneous manifestations. - In late 1958, a smallpox-like illness occurred among a population of cynomolgus monkeys in Copenhagen, leading to the first isolation of monkeypox virus. - No clinical symptoms were observed before the rash phase, which was characterized by a maculopapular eruption. - Between 1960 and 1968, outbreaks occurred among captive monkeys in the United States and the Netherlands, but no human infections were confirmed. - In 1970, the first human case was reported in a 9-month-old boy in the Democratic Republic of the Congo. By 1971, six additional cases had been confirmed in West Africa. - In 2003, the first outbreak outside Africa occurred in the United States through pet prairie dogs infected by rodents imported from Ghana. - After the 2017–2018 outbreak in Nigeria, imported cases were reported in the United Kingdom, Israel, and Singapore in 2018. In May 2022, a new global outbreak, mainly involving men who have sex with men, began primarily in Europe. - Among fatal cases in Nigeria, 4 of 7 patients had HIV infection and were not receiving antiretroviral therapy. - The overall case fatality rate of the West African lineage, corresponding to Clade II, tended to be lower than that of the Central African lineage, corresponding to Clade I. - Epidemiologically, cases in the 1970s and 1980s occurred mainly in young children, whereas during the 2017–2018 Nigerian outbreak, the median age was 29 years. - Nosocomial transmission has also been reported. - The basic reproduction number has been estimated to be 0.6–1.0 for the Central African lineage and lower for the West African lineage. - As of 2022, a total of 71,096 infections had been reported across 107 countries, of which 70,377 occurred in countries with no previous reports of monkeypox. - As of October 4, 2022, 98.5% of cases were male, and men aged 31–40 years accounted for 39.5% of cases. - Transmission outside networks of men who have sex with men has also been reported, although transmission through sexual contact has been suggested. - Reports of multiple sexual partners and lesions around the genital area suggest transmission during sexual activity. - In previous outbreaks, prodromal symptoms such as systemic symptoms and lymphadenopathy were reported; however, the current outbreak has shown several differences, including lesion onset without prodromal symptoms and a mean incubation period of 9.22 days. - Tecovirimat and brincidofovir are the only therapeutic agents currently being used under investigational new drug approval. - Tecovirimat may shorten the duration of viral shedding and hospitalization. - Brincidofovir has been reported to improve survival in animal studies. - Several vaccinia immune globulin products and vaccines have been approved. However, third-generation highly attenuated vaccinia virus vaccines are not currently approved specifically for monkeypox prevention. - MVA-BN has received emergency authorization in several countries. - Some countries have issued recommendations to administer third-generation smallpox vaccines to individuals at increased risk of infection. | ID 6-Ref 6   - Monkeypox is an infection caused by an orthopoxvirus of the family Poxviridae. Clade I is primarily found in Central Africa, whereas Clade II is associated with West Africa. - Although monkeypox is a zoonotic disease, its reservoir remains unknown. Rodents such as forest squirrels and rats are considered possible reservoirs, while non-human primates such as monkeys may serve as intermediate hosts. - In both children and adults, the clinical course consists of an incubation period, with a mean duration of 13 days and a range of 3–34 days, followed by a prodromal phase lasting 1–4 days and an exanthematous phase lasting 2–4 weeks. - The prodromal phase is characterized by symptoms such as high fever, headache, malaise, and lymphadenopathy. - Vesicular lesions develop on the face, trunk, and extremities. Involvement of the palms and soles is an important feature that helps distinguish monkeypox from smallpox. Risk factors for severe disease include childhood, pregnancy, and HIV infection. - Clade II is associated with a lower case fatality rate. Previously, transmission included both animal-to-human and human-to-human spread; however, recent dissemination has been driven mainly by human-to-human transmission, with household transmission also reported. - Sexual transmission is considered uncommon in Africa. - On May 6, 2022, the first case associated with the multinational outbreak was reported in the United Kingdom in a traveler from Nigeria. - Cases were subsequently reported in several countries. Among men whose sexual orientation was known, 96% were men who have sex with men. - The frequent occurrence of lesions around the anus and genital area, the higher incidence among individuals with multiple sexual partners, and the detection of viral DNA in semen suggest an association with sexual activity; however, whether this reflects a change in the mode of transmission remains unclear. - In the current outbreak, many patients did not have a prodromal phase. - Rash was observed in 97% of patients, and perianal lesions were observed in 70%. - Patients with perianal lesions may present with symptoms such as anal pain, proctitis, and tenesmus. - Laryngitis and oral lesions may also occur. - The case fatality rate of Clade II monkeypox is less than 1%. Deaths among young individuals without immunodeficiency have been suspected to be associated with encephalitis, although this remains under investigation. - In the United States, tecovirimat has been approved as a therapeutic agent for smallpox, but its use for monkeypox is currently investigational. - Further evaluation, including randomized controlled trials, is needed. Monoclonal antibodies are currently under development. - Currently available vaccines include ACAM2000 and MVA-BN. - MVA-BN was adopted as a smallpox vaccine and was approved as a monkeypox vaccine in 2019. In the United Kingdom and France, vaccination is recommended for men at high risk. - Monkeypox can be prevented by smallpox vaccination; however, since routine smallpox vaccination was discontinued in 1980, population immunity has declined, creating a risk of outbreaks. - Declining population immunity may partly explain the current outbreak, but it is likely to remain a persistent issue. - It is particularly important to prevent spread through education of high-risk groups and early case detection, to establish rapid diagnostic methods, and to evaluate strategies for the use of currently available treatments and vaccines. |
| ID 9-Ref 6   - In 1958, a smallpox-like disease occurred among a population of cynomolgus monkeys in Copenhagen, leading to the first isolation of monkeypox virus. - The reservoir of monkeypox virus remains unknown, although rodents such as squirrels are considered possible reservoirs. - The Democratic Republic of the Congo has been the most affected country. In previous outbreaks, infections were common among children, whereas outbreaks since 2017 have predominantly involved adults, with frequent household transmission. - Between 1960 and 1968, outbreaks occurred among captive monkeys in the United States and the Netherlands, but no human infections were confirmed. - In 1970, the first human case was reported in a 9-month-old boy in the Democratic Republic of the Congo. By 1971, six additional cases had been confirmed in West Africa. - In 2003, the first outbreak outside Africa occurred in the United States through pet prairie dogs infected by rodents imported from Ghana. - After the 2017–2018 outbreak in Nigeria, imported cases were reported in the United Kingdom, Israel, and Singapore in 2018. In May 2022, a new global outbreak began, mainly involving men who have sex with men, particularly in Europe. - Although men who have sex with men account for a large proportion of infected individuals, outbreaks unrelated to sexual orientation have also occurred. - Monkeypox has three clinical phases and is generally self-limiting, although it may result in sequelae. - Most patients develop prodromal symptoms such as fever, but rash may appear without preceding prodromal symptoms. The rash is variable and is often observed around the anus and genital area. - For treatment, tecovirimat has been approved in the United States and Europe, and brincidofovir has been approved in the United States. - Two types of live vaccines are available, and third-generation vaccines are recommended for high-risk men in some regions. | ID 7-Ref 6   - This article is a review of the epidemiology, virology, treatment, and prevention of mpox. - Mpox virus was first isolated and identified in Copenhagen in 1958. - The first human infection was identified and reported in Africa in 1970. - Since 2003, imported cases have been reported outside Africa, including in the United States and the United Kingdom. In particular, in 2022, many cases were reported among men who have sex with men, mainly through sexual contact. - Mpox is caused by a DNA virus belonging to the genus Orthopoxvirus and is closely related to variola virus. It causes rash, skin lesions, and lymphadenopathy. - Based on genetic background, mpox virus is classified into three clades. - According to the new classification, Clade 1 corresponds to the Central African lineage, Clade 2 to the West African lineage, and Clade 3 to viruses associated with outbreaks in Europe and the Americas. - Mpox is a zoonotic infection and can be transmitted through contact with body fluids or lesions of infected animals, such as rabbits and prairie dogs. - Human-to-human transmission may occur through contact with body fluids or skin lesions, or through contaminated bedding. - Clinically, the mean incubation period is 13 days, and skin lesions appear after prodromal symptoms such as fever and headache. - During the 2022 outbreak among men who have sex with men, genital and pharyngeal lesions were frequently reported. - Epidemiologically, the number of cases has been increasing, particularly in Africa. - In 2022, more than 98% of reported cases occurred in men, especially men who have sex with men. - Children, pregnant women, and patients with HIV infection are more likely to develop severe disease, and cases caused by Clade 1 are associated with a higher mortality rate. - Therapeutic options include tecovirimat and brincidofovir. - In refractory cases, administration of immunoglobulin may be considered. - Available vaccines include ACAM2000 and MVA-BN, which may be used for post-exposure prophylaxis and vaccination of high-risk individuals. |
| ID 10-Ref 6   - In 1958, a smallpox-like disease occurred among a population of cynomolgus monkeys in Copenhagen, leading to the first isolation of mpox virus. - In 1970, the first human case was reported in a 9-month-old boy in the Democratic Republic of the Congo. - In 2003, the first outbreak outside Africa occurred in the United States through pet prairie dogs infected by rodents imported from Ghana. - After the 2017–2018 outbreak in Nigeria, imported cases were reported in the United Kingdom, Israel, and Singapore in 2018. In May 2022, a new global outbreak began, mainly involving men who have sex with men, particularly in Europe. - Tecovirimat and brincidofovir are currently used as therapeutic agents and have shown some efficacy. Vaccines have also been approved for use in high-risk individuals. - Future priorities are clear: education to prevent infection and transmission, development of tests for rapid and accurate diagnosis, and evaluation of currently available treatments and vaccines. |  |
| ID 11-Ref 6   - In 1958, a smallpox-like disease occurred among a population of cynomolgus monkeys in Copenhagen, leading to the first isolation of monkeypox virus. - Between 1960 and 1968, outbreaks occurred among captive monkeys in the United States and the Netherlands, but no human infections were confirmed. - In 1970, the first human case was reported in a 9-month-old boy in the Democratic Republic of the Congo. By 1971, six additional cases had been confirmed in West Africa. - In 2003, the first outbreak outside Africa occurred in the United States through pet prairie dogs infected by rodents imported from Ghana. - After the 2017–2018 outbreak in Nigeria, imported cases were reported in the United Kingdom, Israel, and Singapore in 2018. In May 2022, a new global outbreak began, mainly involving men who have sex with men, particularly in Europe. - Human-to-human transmission occurs mainly through contact with biological fluids or infected skin lesions of patients. In Africa, the source of transmission is often unknown, and the relative contributions of human-to-human and animal-to-human transmission vary substantially across studies and remain unclear. - Among 10,729 male patients whose sexual orientation was known, 10,300, or 96.0%, self-identified as men who have sex with men. Although transmission appears to be occurring within sexual networks of men who have sex with men, there is also evidence of transmission beyond these groups. - Overall, 71.4% of patients presented with systemic symptoms, and 49.0% had localized lymphadenopathy. In addition, 70.5% had anogenital cutaneous and mucosal lesions, while 7.0% had oral cutaneous and mucosal lesions. - Vaccine development is ongoing, and MVA-BN has received emergency authorization from health authorities in several countries, including France. It is being used during outbreaks for contacts at high risk of infection. - Some countries, including the United Kingdom and France, recommend administration of a third-generation smallpox vaccine to men at high risk of infection. - Moving forward, three priorities will be important: strengthening education on infection prevention among high-risk populations, developing diagnostic testing methods, and evaluating and developing vaccines. |  |
| ID 12-Ref 7   - A case–control study was conducted across 12 jurisdictions in the United States to evaluate the effectiveness of the JYNNEOS vaccine in preventing mpox. - The study included men who have sex with men and transgender adults aged 18–49 years. During the period from April 19, 2022, to March 31, 2023, 309 vaccinated individuals were compared with 608 control individuals. - The adjusted vaccine effectiveness was approximately 75% after one dose and approximately 86% after two doses, demonstrating a high level of protection. - Sufficient effectiveness was confirmed across all routes of administration, including both subcutaneous and intradermal vaccination. | ID 14-Ref 7   - As of March 2023, more than 30,000 mpox cases had been reported in the United States, affecting men who have sex with men and other populations. - The JYNNEOS vaccine was approved by the FDA in 2019 as a subcutaneous vaccine for the prevention of smallpox and mpox. - Vaccination was administered both to persons with confirmed or presumed contact with patients as post-exposure prophylaxis and to persons at risk for mpox as pre-exposure prophylaxis. - A matched case–control study was conducted across U.S. jurisdictions to evaluate vaccine effectiveness against mpox among men who have sex with men and transgender persons aged 18–49 years. - During the period from August 19, 2022, to March 31, 2023, the study included 309 case patients and 608 control participants. - The adjusted vaccine effectiveness was 75.2% after one dose (95% CI, 61.2%–84.2%) and 85.9% after two doses (95% CI, 73.8%–92.4%). - The adjusted vaccine effectiveness of two doses by route of administration was 88.9% for subcutaneous vaccination (95% CI, 56.0%–97.2%), 80.3% for intradermal vaccination (95% CI, 22.9%–95.0%), and 86.9% for heterologous administration (95% CI, 69.1%–94.5%). - Among immunocompromised participants, the adjusted effectiveness of two doses was 70.2% (95% CI, −37.9% to 93.6%). - JYNNEOS was effective in reducing the risk of mpox; however, the difference in duration of protection between one and two doses remains unclear. - Persons at high risk should receive two doses. - A questionnaire survey was also conducted among sexually active men who have sex with men or transgender persons aged 18–49 years, including both patients diagnosed with mpox and those without mpox. - Compared with control participants, case patients were more likely to be non-Hispanic Black or African American (27.2% vs. 16.9%) or Hispanic or Latino (32.4% vs. 23.4%). Compared with HIV-negative participants, HIV-positive participants were significantly more likely to have experienced homelessness, transactional sex, and HIV infection. - Case patients were also more likely to be immunocompromised and to have a known history of contact with a person with mpox. - Regarding vaccination status, 22.5% had received two doses, 32.2% had received one dose, and 45.4% were unvaccinated. The adjusted vaccine effectiveness was 75.2% after one dose and 85.9% after two doses. - These findings are consistent with previous reports and support the use of JYNNEOS for the prevention of mpox. - The overlap in confidence intervals for vaccine effectiveness estimates suggested no clear difference according to immunocompromised status. - Additional preventive measures should be considered, particularly for immunocompromised persons. - Two-dose vaccination is recommended regardless of the route of administration, although the duration of protection requires further evaluation. |
| ID 13-Ref 7   - A case–control study was conducted across 12 jurisdictions in the United States to evaluate the effectiveness of the JYNNEOS vaccine in preventing monkeypox. - The study included men who have sex with men and transgender adults aged 18–49 years, comparing 309 case patients with 608 control participants. - The adjusted vaccine effectiveness was approximately 75% after one dose and approximately 86% after two doses, demonstrating a high level of protection. - Sufficient effectiveness was confirmed across all routes of administration, including subcutaneous vaccination and dose-sparing intradermal vaccination. - Among immunocompromised individuals, vaccine effectiveness was 51.0% after partial vaccination and 70.2% after full vaccination. Among immunocompetent individuals, vaccine effectiveness was 72.1% after partial vaccination and 87.8% after full vaccination. - Although no significant difference was observed according to immunocompromised status, vaccine effectiveness tended to be lower among immunocompromised individuals, suggesting a potentially weaker immune response. | ID 15-Ref 7   - As of March 31, 2023, more than 30,000 cases of monkeypox (mpox) had been reported in the United States, particularly affecting gay, bisexual, and other men who have sex with men, as well as transgender persons. - In 2019, the JYNNEOS vaccine was approved by the FDA as a two-dose subcutaneous series, administered as 0.5 mL per dose 4 weeks apart, for the prevention of smallpox and mpox. Subsequently, on August 9, 2022, the FDA issued an Emergency Use Authorization for a dose-sparing two-dose intradermal regimen of JYNNEOS, administered as 0.1 mL per dose 4 weeks apart. - To evaluate the effectiveness of the JYNNEOS vaccine, a matched case–control study was conducted among men who have sex with men and transgender adults to assess vaccine effectiveness against mpox. - The adjusted vaccine effectiveness was 75.2% for partial vaccination, defined as one dose (95% confidence interval, 61.2%–84.2%), and 85.9% for full vaccination, defined as two doses (95% confidence interval, 73.8%–92.4%). - The adjusted vaccine effectiveness of full vaccination by route of administration was 88.9% for subcutaneous administration (95% CI, 56.0%–97.2%), 80.3% for intradermal administration (95% CI, 22.9%–95.0%), and 86.9% for heterologous administration (95% CI, 69.1%–94.5%). These findings suggest that vaccination via any of these routes may provide comparable protection against mpox. - Among immunocompromised patients, the adjusted vaccine effectiveness after full vaccination was 70.2% (95% CI, −37.9% to 93.6%), whereas among immunocompetent patients it was 87.8% (95% CI, 57.5%–96.5%). - Further studies are needed to evaluate the duration of protection, which may differ according to the number of doses received and the route of administration. - At present, JYNNEOS vaccination coverage remains low among persons at risk for mpox, and many eligible individuals have not completed the two-dose series. Further promotion of vaccination among at-risk populations is warranted. |
|  | ID 16-Ref 7   - By March 31, 2023, more than 30,000 cases of mpox had been reported in the United States, disproportionately affecting gay, bisexual, and other men who have sex with men, as well as transgender persons. - The JYNNEOS vaccine, administered as a two-dose subcutaneous series 4 weeks apart, was approved by the FDA in 2019 for the prevention of smallpox and mpox. - JYNNEOS vaccination was offered to the following groups: - Post-exposure prophylaxis: Persons with known or possible contact with a patient with mpox. Post-exposure prophylaxis is most effective when administered as soon as possible, ideally within 4 days after exposure. When administered 4–14 days after exposure, it may not completely prevent disease onset but may reduce disease severity. - Pre-exposure prophylaxis: Persons at increased risk of mpox infection, including those with specific risk factors or behaviors associated with increased risk. - The vaccine has been shown to induce similar immune responses when administered intradermally or subcutaneously. This allowed broader vaccine access for persons at high risk of exposure and those requiring early post-exposure intervention, thereby helping to control the spread of mpox. - However, data on vaccine effectiveness were limited, and a case–control study was conducted at 12 sites in the United States. - The objective of the study was to evaluate vaccine effectiveness against mpox among men who have sex with men and transgender persons aged 18–49 years. - During the period from August 19, 2022, to March 31, 2023, 309 case patients and 608 control participants were evaluated. The adjusted vaccine effectiveness was 75.2% among persons who had received one dose and 85.9% among those who had received two doses. Among patients with immunocompromising conditions, vaccine effectiveness was 70.2% and 87.8%, respectively. - JYNNEOS vaccination appears to be effective; however, the duration of protection after one versus two doses remains unclear. For persons at high risk, completion of the two-dose series is recommended. |
| ID 20-Ref 8   - This review summarizes the extent to which human-to-human respiratory transmission of mpox, formerly known as monkeypox, occurs. - Animal studies have shown that infection via the respiratory route is possible, and limited evidence of respiratory transmission between animals has also been reported. - Environmental sampling studies have reported the detection of MPXV in the air. However, in actual outbreaks, close contact appears to be the main route of transmission, and no cases have definitively demonstrated respiratory transmission. - Respiratory symptoms and the presence of virus in the upper respiratory tract suggest the possibility of respiratory transmission, but they do not provide conclusive evidence of such transmission. - Environmental sampling studies have shown the presence of virus in air and on surfaces, but this does not necessarily indicate infectivity or human-to-human transmission. Therefore, no definitive conclusion can be drawn at present. - At present, the risk of human-to-human respiratory transmission is considered low; however, continued research and surveillance are needed. - Based on the above, the UKHSA and CDC currently recommend that patients with mpox wear an appropriate medical mask when close contact with others cannot be avoided. They also recommend that persons in contact with patients with mpox wear respiratory protection or an appropriate medical mask when contact with an infected person extends beyond brief interaction. | ID 17-Ref 8   - The route of respiratory transmission of mpox remains unclear. - Therefore, the authors examined evidence regarding respiratory transmission of mpox virus (MPXV) based on animal models, human outbreak investigations, case reports, and environmental studies. |
|  | ID 18-Ref 8   - Data on respiratory transmission of mpox were collected from animal models, human outbreak investigations and case reports, and environmental studies. - Reports included animal-to-animal transmission via the respiratory route and detection of mpox droplet nuclei in environmental samples. - Although human outbreak reports suggest contact-associated transmission, there is currently no clear evidence of respiratory transmission in humans. - Based on currently available reports, human-to-human transmission of mpox via the respiratory route appears unlikely; however, continued evaluation of this possibility is warranted. |
|  | ID 19-Ref 8   - In 2022, infections caused by clade II mpox virus occurred worldwide. - Factors that may have contributed to the outbreak included transmission through networks of men who have sex with men, waning immunity from smallpox vaccination, and increased testing for the virus. - Possible routes of transmission include percutaneous exposure, direct contact, and inhalation of particles. The current outbreak, in which anogenital lesions are common, suggests sexual transmission as a major route. - Respiratory transmission may occur through inhalation of particulate matter derived from oral and extraoral lesions, as suggested by animal studies and PCR testing of swabs from infected patients; however, its relevance remains unclear. - Upper and lower respiratory tract symptoms have been reported in humans, but they were uncommon during the 2022 outbreak. - Previous studies have suggested multiple possible routes of transmission. - During a flight in 2021, masks were worn because of the COVID-19 pandemic, and this may have helped prevent respiratory transmission. - In the 2022 outbreak, no secondary transmission was reported in settings involving aerosol-generating procedures or in childcare facilities such as schools. - Clinical and outbreak data indicate that transmission occurred primarily through close and prolonged contact. - Several reports suggest that the virus can remain viable on surfaces for prolonged periods; however, there are three possible routes of transmission, and it remains unclear whether transmission occurred via the respiratory route. - Several reports have demonstrated the presence of virus in air and on surfaces, but this does not necessarily indicate human-to-human respiratory transmission. If respiratory transmission were a major route, more extensive spread would likely have been observed. - Accumulated data support a minimal role for respiratory transmission, although continued evaluation is needed because of ongoing viral evolution. - APOBEC3 activity has been suggested to have played an important role in the evolution of clade II MPXV. - Public health authorities recommend mask use and basic preventive measures when contact with patients with mpox cannot be avoided. |

* In all articles, we verified that the accuracy of the meanings (not verbatim translation) provided in the documents was consistent between the “with LLM” group and the “without LLM” group.

Abbreviations: ID, sequential identifier assigned to each summary; Ref, reference number of the source article in the main manuscript as below:

1. Smith TG, Gigante CM, Wynn NT, et al. Tecovirimat resistance in mpox patients, United States, 2022-2023. Emerg Infect Dis, 2023 ; 29: 2426-2432.
2. Gessain A, Nakoune E, Yazdanpanah Y. Monkeypox. N Engl J Med, 2022 ; 387: 1783-1793.
3. Dalton AF, Diallo AO, Chard AN, et al. Estimated effectiveness of JYNNEOS vaccine in preventing mpox: a multijurisdictional case-control study - United States, August 19, 2022-March 31, 2023. MMWR Morb Mortal Wkly Rep, 2023 ; 72: 553-558.
4. Beeson A, Styczynski A, Hutson CL, et al. Mpox respiratory transmission: the state of the evidence. Lancet Microbe, 2023 ; 4: e277-e283.
